# Social-cognitive barriers to ethical authorship

**DOI:** 10.3389/fpsyg.2015.00877

**Published:** 2015-07-21

**Authors:** Jordan R. Schoenherr

**Affiliations:** ^1^Department of Psychology, Carleton UniversityOttawa, ON, Canada; ^2^Department of Innovation in Medical Education, University of OttawaOttawa, ON, Canada

**Keywords:** research misconduct, research integrity, inappropriate authorship, source credibility, applied ethics

## Introduction

The apparent increase in research misconduct in the scientific literature has caused considerable alarm in both the biomedical (Benos et al., 2005; Smith, 2006) and psychological research communities (Stroebe et al., 2012). An understanding of research misconduct must be informed by the recognition that the norms of science might be quite general (e.g., Merton, 1942; Bronowski, 1965), ambiguous (Cournand and Meyer, 1976), or even contradictory (e.g., Mitroff, 1974; Ziman, 2000), leading to possible disagreements in terms of what constitutes misconduct within a research community (Fields and Price, 1993; Berk et al., 2000; Al-Marzouki et al., 2005). Considerable insight can be gained from research on behavioral ethics (e.g., Bazerman and Tenbrunsel, 2011; Ariely, 2012; Greene, 2013). Using inappropriate authorship practices as an illustrative example, I consider the role of social-cognitive mechanisms in research misconduct while also suggesting preventative measures.

## Prevalence of research misconduct

Widespread interest in dishonesty in research began comparatively recently in the history of the sciences (e.g., Broad and Wade, 1982; Steneck, 1999) although there was an early recognition that misconduct was a feature of scientific research (Babbage, 1830). Though a definitive set of forms of misconduct has yet to be identified, fabrication, falsification, and plagiarism (FFP) are generally cited as clear violations of scientific norms. In a review of studies of FFP, Steneck (2006) estimated that its occurrence rate fell within a range of 1.0 and 0.001% (for recent support, see Fanelli, 2009). He further suggested that research practices reflect a normal distribution, with FFP representing outlying behaviors. More ambiguous behaviors, or questionable research practices (QRP), have a much higher rate of occurrence, with Steneck suggesting that they constitute 10–50% of all research practices. QRPs represent an interesting form of misconduct in that they apparently reflect a feature of normal science (De Vries et al., 2006) thereby suggesting that they might reflect the social-cognitive processes underlying the dishonest behaviors of people more generally (e.g., Bazerman and Tenbrunsel, 2011; Ariely, 2012).

Inappropriate authorship practices are a prevalent form of QRP. For instance, they can represent a failure to recognize an original contribution to research (*ghost authorship*) or a misattribution of the research to those who have not contributed (*gift authorship*). The prevalence of inappropriate authorship practices is reflected in studies conducted by Flanagin et al. (1998) and Wislar et al. (2011) wherein they observed a decrease in the prevalence of ghost authorship from 11.5 to 7.9% between 1996 and 2008. In contrast, the number of articles affected only by gift authorship remained relative constant with a non-significant decrease from 19.3 to 17.6% during the same period (for similar findings, see Mowatt et al., 2002; Mirzazadeh et al., 2011; cf. Stretton, 2014). Accounting for the stability and change of inappropriate authorship practices represents an important task for applied ethics as the assignment of credit can lead to stratification within the scientific community (e.g., Cole and Cole, 1973).

## The social cognition of credit and credibility

Early commentators attributed research misconduct to a range of factors including publication pressure, competition, and psychopathy (Chubin, 1985; cf. Braxton and Bayer, 1994). However, the prevalence of QRP suggests that more general social-cognitive mechanisms can account for research misconduct. Analyses of cases of misconduct have suggested a number of contributing factors (for a review, see Davis et al., 2007). Here I will consider how inappropriate authorship practices can be understood in terms of influence of social conventions and conformity, the reciprocity norms of exchange systems, as well as role schemata and status.

### Social conventions and conformity bias

The social conventions and ethical norms of science are evidenced in its cultural, structural, and organizational systems (Davis, 2003). Empirical support for the role of social conventions in judgements of ethical conduct comes from a number of sources. Kohlberg (1976) outlines a model with three stages of moral reasoning. A preconventional stage of moral reasoning defined by self-interest is contrasted against a subsequent stage of conventional moral reasoning wherein social norms of the group or society are used to judge behavior. While an additional post-conventional stage relies on the use of ethical principles, Kohlberg found that few individuals achieve this stage of reasoning (cf. Rest et al., 1999). Even when morals can be clearly identified, conventions play an important role in social interactions (Turiel, 2002) with conformity biases maintaining cultural norms (e.g., Whiten et al., 2005; Efferson et al., 2008). Experimental evidence also suggests that dishonest behaviors increase when in-group members are observed to engage in these behaviors (Gino et al., 2009).

Studies of academic misconduct have also demonstrated the influence of conventions and conformity, in terms of peer influence on cheating. In their study, McCabe and Treviño (1997) found that peer behavior and fraternity/sorority membership were positively related to the occurrence of misconduct, whereas perceived peer disapproval was negatively related to the occurrence of misconduct (see also, McCabe et al., 2001). Social conventions additionally offer an explanation for the difficulty in implementing successful ethics training programs, with disciplinary and departmental values being associated with researcher behavior (e.g., Anderson et al., 1994) and regression from post-conventional reasoning to conventional reasoning (Rennie and Rudland, 2002; Hren et al., 2011).

### Social organization and reciprocal exchange

The nature and prevalence of dishonesty can also be understood in terms of the norms of social exchange systems (e.g., Fiske, 1991). Fiske (1991) considers four kinds of exchange systems that differ in terms of the commensurability of the objects in the reciprocal exchange relationship (*equality matching*; *communal sharing*; *market pricing*; and *authority ranking*). These systems will in turn determine what is seen as honest and dishonest behavior. For instance, a researchers' contributions to a research project (e.g., theory, data collection, statistics) might be deemed unique and incommensurable, making judgments of proportion of credit arbitrary (*communal sharing*) or exceedingly difficult (*equality matching*). Researchers might instead assume that contributions can be differentiated and are quantifiable in terms of an absolute value that can be used to assign a proportion of authorship credit and responsibility (*market pricing*). Rightly or wrongly, this exchange norm appears to underscore the belief that the order of authorship reflects the proportion of contribution a researcher has made to a study (e.g., ICJME, 2005/2008). Finally, researchers might assume that authority should be the primary determinant of the assignment of credit (*authority ranking*), something that I will return to the next section.

Scientific research has been defined as an exchange system by a number of authors. Hagstrom (1982) suggested that a research article can be viewed as analogous to a gift whereas Street et al. (2010) have noted that “journal articles are valuable intellectual property,” (p. 1458). These observations as well as others suggest that reciprocity can exert considerable influence on our judgements (Gouldner, 1960; Fiske, 1991). In terms of authorship, credit might be given due to the need for reciprocity by junior researchers receiving funding or advice from senior researchers. Authorship deals, or “mutual support authorships,” wherein researchers include names of authors so as to have their name included on a project, also explicitly reflect an overt reciprocity strategy (Claxton, 2005; Louis et al., 2008). In addition to overt pressure, “lab chiefs” might be assigned undue credit as a result of researchers receiving career advice and financial support thereby enabling the research process while not directly contributing to intellectual content of a specific publication (Broad and Wade, 1982; Claxton, 2005; Street et al., 2010). Similarly, the provision of sponsorship might be perceived as sufficient grounds for receiving authorship (Louis et al., 2008). Both of these behaviors might be best understood in terms of the *halo effect* (Thorndike, 1920; Nisbett and Wilson, 1977) wherein participants overgeneralize from one attribute to the individual as a whole (see also, Harvey et al., 2010).

### Source credibility, status, and role schemata

Due to the need to allocate limited attention, researchers must identify a subset of individuals that appear to provide credible information (Thorngate et al., 2011). Source credibility exerts considerable influence in the formation and change of attitudes (e.g., Petty et al., 1997). Thus, the contributions of researchers who are deemed to have greater credibility *a priori* might not be judged as critically as those with less credibility. Supporting this, studies that manipulate power (e.g., Guinote, 2013) have demonstrated that those in comparatively powerless position have reduced attention and short-term memory resources due to a need to respond to those in positions of power. In comparison, those in powerful positions are more likely to engage in confirmation bias in the pursuit of their goals. Collaborations between senior and junior researchers will likely be influenced by these situational factors (e.g., Sullivan and Ogloff, 1998) making it harder for junior members to assess the contributions of senior authors. Gift authorship can also be understood as an instance of a desire to confer credibility onto a research project. Peters and Ceci (1982) demonstrated this influence in a quasi-experiment wherein journal articles previously published by prestigious authors were resubmitted with fictitious non-prestigious names. When submitted with non-prestigious names, the majority of referees rejected these previously accepted articles.

The effects of source credibility can also be understood in terms of status assigned to social roles (e.g., Merton, 1968; Azoulay et al., 2014). Role schemata contain information pertaining to behaviors and obligations associated with a given role in a particular social context, thereby influencing the behavior and judgments of self and others. Historically, Shapin (1989) has noted that despite significant intellectual contributions to the design and conduct of experiments, technicians were not deemed to warrant authorship. As noted above, lab chiefs also appear to be awarded undue credit (Broad and Wade, 1982) and this might be attributed to perceived differences in credibility. If students and other personnel associated with a research project are believed to have a “supporting” role, their contributions might not be attributed to them. Rather, they might need to be legitimated by credible others in order for them to be accepted within a research community. More generally, authority ranking exchange systems assume that those in positions of authority are deemed to warrant more resources (Fiske, 1991). This would manifest itself as being awarded a disproportionate amount of credit. However, role schemata can also benefit those perceived to be in a subordinate position. As Zuckerman (1968) observed, Nobel laureates often appear to have awarded greater authorship credit to less prestigious collaborators. Moreover, those with higher status have also been found to express more favorable attitudes toward preserving the ethical norms of their discipline (e.g., Braxton and Bayer, 1994).

## Conclusions

If inappropriate authorship practices can be accounted for by general social-cognitive processes, then an ameliorative program at least appears possible in principle. In opposition to these efforts, ethics training programs developed in an applied context have not always been successful (e.g., Brown and Kalichman, 1998; Fisher et al., 2009). Such failures likely stem from an ethical “fudge factor,” a failure to attend to ethical norms on a moment-to-moment basis, and the observation of dishonest behavior of peers (e.g., Bazerman and Tenbrunsel, 2011; Ariely, 2012; Greene, 2013). Indeed, rather than engaging in an explicit reasoning process (Kohlberg, 1976; Rest et al., 1999) our responses to ethical dilemmas often appear to be automatic (Haidt, 2007) and are susceptible to loss framing and time pressure (e.g., Kern and Chugh, 2009). Together with self-deception and justifications (Tenbrunsel and Messick, 2004; Shalvi et al., 2011), ethical facets of authorship decisions might become less salient. Reciprocity norms, along with the “publish or perish” framing of contemporary academic publishing, would certainly support these behaviors. These enablers must be acknowledged and addressed if we hope to reduce ghost and gift authorship.

Having recognized the influence of social context and automaticity, three general proposal appear to offer promise to reduce the prevalence of unethical behaviors. First, we must ensure that researchers are aware of the ethical standard and norms of authorship within their research community and that co-authors discuss expectations and roles throughout the research process. Standards such as those provided by the ICJME (2005/2008) are useful points of reference for the assignment of authorship/contributorship. Second, by continually priming these norms with ongoing discussions at departmental and disciplinary levels, we are likely to obtain similar reductions in dishonest behavior as those observed in laboratory studies (Mazar et al., 2008). Finally, to disincentivize dishonest behavior stemming from a “publish or perish” academic culture, we must consider adopting criterion for hiring, promotion, and funding decisions based on the quality of a restricted number of publications rather than the total number of publications produced by an individual.

## Funding

This research was supported by funding from the Ottawa Health Research Institute.

### Conflict of interest statement

The authors declare that the research was conducted in the absence of any commercial or financial relationships that could be construed as a potential conflict of interest.
